# Recurrent Hiatal Hernia Resulting in Rightward Mediastinal Shift: Diagnostics in Cardiology and Clinical Pearls

**DOI:** 10.7759/cureus.16521

**Published:** 2021-07-20

**Authors:** Divy Mehra, Javier Alvarado, Yanet Diaz-Martell, Lino Saavedra, James Davenport

**Affiliations:** 1 Ophthalmology, Nova Southeastern University Dr. Kiran C. Patel College of Osteopathic Medicine, Fort Lauderdale, USA; 2 Internal Medicine, Kendall Regional Medical Center, Kendall, USA; 3 Cardiology, Kendall Regional Medical Center, Kendall, USA

**Keywords:** dextrocardia, dextroposition, electrocardiogram, hiatal hernia, cardiac compression

## Abstract

On radiographic imaging, the finding of a right-sided heart location can be due to multiple etiologies and may be congenital or acquired. We present the case of a 71-year-old male with a self-reported past medical history of hiatal hernia and previously diagnosed dextrocardia. The patient experienced cardiovascular intervention following an ST-elevation myocardial infarction. In the cardiac workup, a low-voltage normal electrocardiogram confirmed dextroposition of the heart due to significant herniation of gastric contents into the thoracic cavity. This gentleman had presumably been diagnosed with dextrocardia, a right-left reversal of heart anatomy and electrophysiology, based on imaging and incomplete workup.

Dextroposition refers to a rightward shift of the mediastinum with no changes in orientation of cardiac anatomy, and therefore unchanged directional orientation of conduction. This is an important distinction from dextrocardia, a mirror-image reversal of the cardiac chambers and heart location in the chest wall, such as that due to congenital ciliary dysfunction. A sliding hernia is an uncommon cause of the rightward mediastinal shift, with few such cases documented in the literature, and cardiovascular manifestations of hiatal hernias are discussed.

This case exemplifies the role of an electrocardiogram in distinguishing between dextrocardia and dextroposition for accurate diagnosis and management.

## Introduction

On imaging, the finding of a right-sided heart location can be due to multiple etiologies and may be congenital or acquired. Dextrocardia, or situs inversus, is a mirror-image reversal of the cardiac chambers and heart location in the chest wall, most commonly diagnosed using a chest radiograph and electrocardiogram for confirmation. Dextroposition is the rightward shift of the heart without inverse reversal of the heart chambers and anatomical formation, which may result from physical compression of herniated gastric contents. Hiatal hernia, with accompanying gastroesophageal reflux disease, is often in the differential in patients presenting to the emergency department with nonspecific chest pain or dyspnea. While these symptoms frequently prompt a full cardiac workup, this pain may be due to visceral esophageal pain or as a result of physical compression of cardiac and respiratory structures in the thoracic cavity by herniated contents. This case details a patient with a long-standing untreated type 1 hiatal hernia that had previously been diagnosed with dextrocardia, later revealed to be a dextroposition through further imaging and workup.

## Case presentation

We present the case of a 71-year-old male with a self-reported past medical history of dextrocardia, hiatal hernia, and recent ST-elevation myocardial infarction status post stent placement in the proximal-mid left anterior descending artery. The patient presented to the emergency department with a chief complaint of generalized weakness and fatigue, and signs and symptoms of hypotension and bradycardia. An electrocardiogram (EKG), chest radiography, echocardiography with 45% ejection fraction, and basic admission labs were completed as a part of an initial cardiac workup, with no pertinent lab irregularities. The patient was given intravenous fluids for volume resuscitation, placed in telemetry with pacer pads, and cardiology was consulted. 

**Figure 1:**
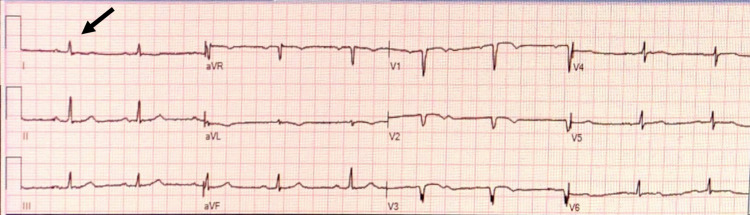
Patient EKG This electrocardiogram (EKG) was taken prior to pacemaker placement in the patient. Findings were read as normal sinus rhythm with low voltage throughout, particularly of QRS waves (arrow denotes a normal but low-voltage QRS complex in Lead 1).

The cardiology team diagnosed the patient with sick sinus syndrome with an indication for pacemaker placement, but upon reviewing the chest radiograph and EKG findings (Figure 1), they could not confirm a diagnosis of dextrocardia. 

**Figure 2:**
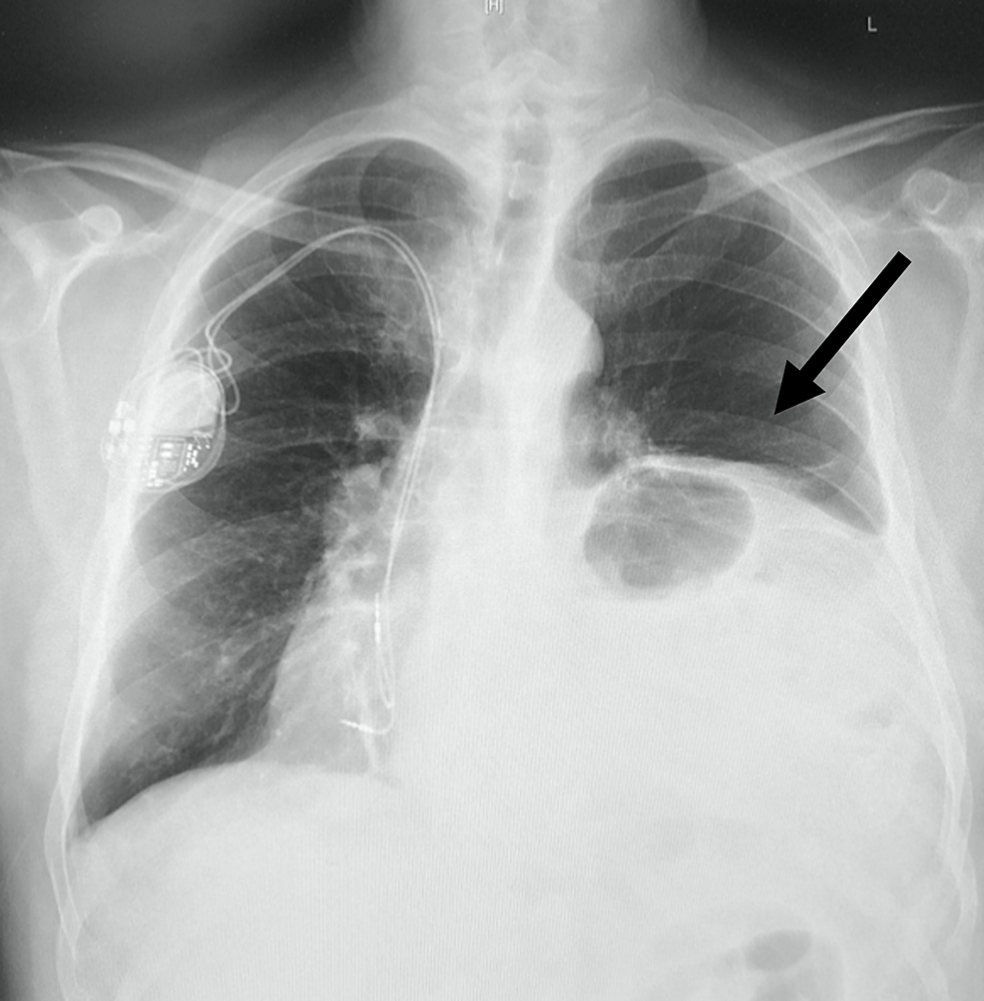
Anterior-Posterior Chest Radiograph This anterior-posterior chest radiograph demonstrates a marked rightward mediastinal shift and asymmetric elevation of the left hemidiaphragm with presumed voluminous hiatal hernia (arrow denotes the herniated abdominal contents in the left thoracic cavity), with pacemaker placement in the right chest wall.

**Figure 3:**
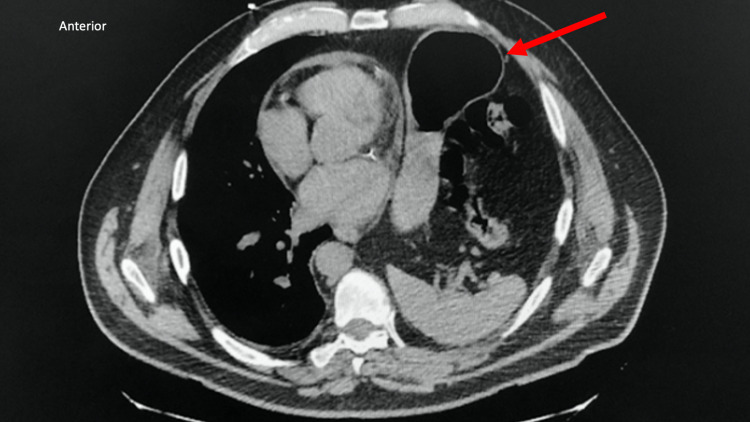
Thoracic CT Scan This transverse chest computerized tomography (CT) scan shows rightward mediastinal shift, coronary calcifications and stents, the elevation of the left hemidiaphragm, herniation of gastric contents into the thoracic cavity (arrow denotes the abnormal presence of abdominal contents, indicated by air-fluid levels, in the left thoracic cavity) with compressive atelectasis of the left lower lung, and lack of mediastinal adenopathy.

The patient underwent the procedure to have a dual-chamber permanent pacemaker implanted in his right chest wall, and the procedure was tolerated well without any complications. Radiographic images (Figure 2 and Figure 3) and fluoroscopic imaging revealed the patient did not have dextrocardia as mentioned in his past medical history. The patient was determined to have a large hiatal hernia that had been displacing his heart towards the extreme right side of his chest wall cavity, demonstrating an entirely right-sided cardiac silhouette mimicking dextrocardia. The patient was later informed he did not have dextrocardia; instead, the patient was educated on his heart’s dextroposition and etiology and thereafter managed for a significant hiatal hernia given symptomatic complaints.

## Discussion

A type 1 hiatal hernia, also known as sliding hiatal hernia, can rarely manifest with cardiac symptomatology when the area of herniation is sufficiently large due to physical compression of the left heart structures, including the left atria, ventricles, or pulmonary veins [1]. The presented case demonstrates a severe rightward mediastinal shift due to recurrent compression by the herniating stomach mass into the thoracic cavity.

Dextroposition or Dextrocardia?

The term ‘dextroposition’ refers to a heart with a leftward-pointed apex that has been shifted to the right due to extracardiac causes, such as a prior pneumonectomy, hypoplasia of the right lung, or a diaphragmatic/hiatal hernia with eventration of a hemidiaphragm, as in this patient [2]. In this case, there is generally no anatomic variation in the heart itself. Conversely, ‘dextrocardia’ refers to both a right-sided heart and rightward-pointed apex. Dextrocardia frequently presents with cardiac chamber disarrangement manifesting as situs inversus, or a mirror image reversal of the left and right heart atria/ventricles [3]. The imaging and electrocardiogram findings in this patient are indicative of a dextroposition cardiac abnormality.

In a dextroposition, EKG findings are nonspecific and generally within normal limits, with possible diffuse low voltage waves, as present in this patient.

In patients with a true dextrocardia in situs inversus, EKG can reveal right axis deviation, a negative QRS complex, inverted P and T waves in lead I, biphasic T waves, and progressively decreasing R-wave amplitude from leads V1 to V6 [4]. These findings were notably absent in this patient.

Hiatal Hernias and Cardiothoracic Anatomy

Type 1 hiatal hernias result from weakness of the phrenoesophageal membrane and disruption of the gastroesophageal junction, allowing herniation of the stomach and migration of the gastroesophageal (GE) junction superior to the hiatus [5]. While the gastric fundus is most commonly affected, the entire stomach organ may be impacted with rotation and strangulation. Severe cases involving large herniated contents tend to occur in older individuals, particularly those older than 50, and those with chronically increased intraabdominal pressure, such as excessive vomiting or chronic constipation. These individuals are more likely to experience symptoms of severe heartburn, difficulty swallowing, regurgitation of food, chest or abdominal pain, vomiting, and shortness of breath [6].

Rare documented cases have reported cardiopulmonary symptomatology as a result of compression of the left atrium, left ventricle, left and right pulmonary veins, and left lung lobes from a hiatal hernia [1,7,8]. In cases of type 1 hiatal hernias as the chief etiology, impaired respiratory function, retrosternal chest pain, and chest tightness have been described as primary symptoms that prompted patients' hospital admissions. In addition, elevated N-terminal prohormone of brain natriuretic peptide (NT-BNP) and EKG changes may be found as confusing signs in these cases [8]. While the majority of sliding hernias are asymptomatic, chest pain or dyspnea may be an atypical presenting symptom. 

Type 1 hiatal hernias, whether reducible or non-reducible, account for 95% of all hiatal hernias and are directly associated with gastroesophageal reflux syndrome (GERD). GERD can be complicated by erosive esophagitis, Barrett’s esophagus, and adenocarcinoma of the esophagus. Thus, given a patient's clinical history and presentation, suspicion of a hiatal hernia may prompt definitive diagnosis and further evaluation or treatment. Different modalities to diagnose hiatal hernias are available depending on the severity of the symptoms, these include; barium swallow studies, upper endoscopy, and esophageal manometry. A complete history and a high level of suspicion for a sliding hernia may help minimize misdiagnoses and optimize treatment plans [6]. Medical management is the mainstay therapy of both medical conditions, including the use of proton-pump inhibitors and H-2 blockers, while a laparoscopic surgical repair is indicated for patients with severe symptoms despite optimal medical management with complications such as ulcers, bleeding episodes, or scarring/strictures [7].

## Conclusions

This case demonstrates the importance of a thorough workup prior to diagnosis of dextrocardia in a patient, as well as the role of electrocardiogram findings in distinguishing between dextroposition and dextrocardia. This case also serves to illustrate the long-term complications and cardiac manifestations of an untreated hiatal hernia.
